# 661W Photoreceptor Cell Line as a Cell Model for Studying Retinal Ciliopathies

**DOI:** 10.3389/fgene.2019.00308

**Published:** 2019-04-05

**Authors:** Gabrielle Wheway, Liliya Nazlamova, Dann Turner, Stephen Cross

**Affiliations:** ^1^Centre for Research in Biosciences, University of the West of England, Bristol, Bristol, United Kingdom; ^2^Human Development and Health, Faculty of Medicine, University of Southampton, Southampton, United Kingdom; ^3^Human Development and Health, Southampton General Hospital, Southampton, United Kingdom; ^4^Wolfson Bioimaging Facility, University of Bristol, Bristol, United Kingdom

**Keywords:** retina, photoreceptor, cilia, ciliopathy, cell model, retinitis pigmentosa

## Abstract

The retina contains several ciliated cell types, including the retinal pigment epithelium (RPE) and photoreceptor cells. The photoreceptor cilium is one of the most highly modified sensory cilia in the human body. The outer segment of the photoreceptor is a highly elaborate primary cilium, containing stacks or folds of membrane where the photopigment molecules are located. Perhaps unsurprisingly, defects in cilia often lead to retinal phenotypes, either as part of syndromic conditions involving other organs, or in isolation in the so-called retinal ciliopathies. The study of retinal ciliopathies has been limited by a lack of retinal cell lines. RPE1 retinal pigment epithelial cell line is commonly used in such studies, but the existence of a photoreceptor cell line has largely been neglected in the retinal ciliopathy field. 661W cone photoreceptor cells, derived from mouse, have been widely used as a model for studying macular degeneration, but not described as a model for studying retinal ciliopathies such as retinitis pigmentosa. Here, we characterize the 661W cell line as a model for studying retinal ciliopathies. We fully characterize the expression profile of these cells, using whole transcriptome RNA sequencing, and provide this data on Gene Expression Omnibus for the advantage of the scientific community. We show that these cells express the majority of markers of cone cell origin. Using immunostaining and confocal microscopy, alongside scanning electron microscopy, we show that these cells grow long primary cilia, reminiscent of photoreceptor outer segments, and localize many cilium proteins to the axoneme, membrane and transition zone. We show that siRNA knockdown of cilia genes Ift88 results in loss of cilia, and that this can be assayed by high-throughput screening. We present evidence that the 661W cell line is a useful cell model for studying retinal ciliopathies.

## Introduction

**FIGURE 1:**
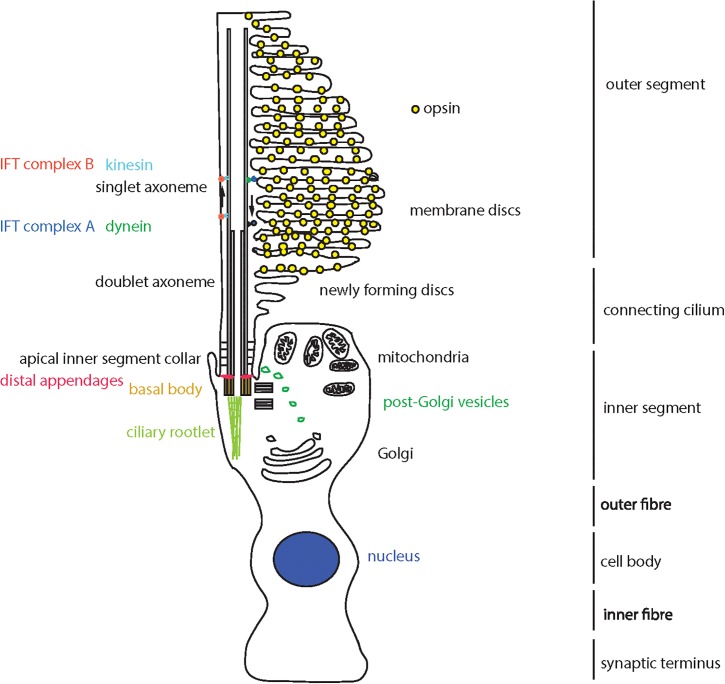
Schematic representation of a cone photoreceptor cell and localization of ciliary proteins. The schematic represents the cone photoreceptor cell outer segment, connecting cilium, inner segment, outer fiber, cell body, inner fiber and synaptic terminus. A number of key components of the ciliary apparatus are color coded and indicated. The IFT complex A (blue) and complex B (red) are represented in the magnified inset.

The sensory primary cilium is an important non-motile cellular organelle, responsible for detecting changes in the extracellular environment and transducing signals to allow the cell to respond accordingly. The retina contains several ciliated cell types, including the retinal pigment epithelium (RPE) and photoreceptor cells. There are two types of photoreceptors; rods and cones, which differ in their shape and the photopigment they contain. Both cell types contain an inner segment, where the nuclei and other organelles are located. Extending from the apical surface of this inner segment is the connecting cilium, which contains a proximal region analogous to the transition zone of primary cilia on other cell types, and a distal region which is unique to the photoreceptor connecting cilia (Dharmat et al., 2018). At the end of the connecting cilium is a huge elaboration of stacks of membrane where the photopigment molecules are located; termed the outer segment (Sjöstrand, 1953). Rods have a long, thin rod-like outer segment containing rhodopsin (Wheway et al., 2014), and cones have a shorter conical outer segment containing opsins which absorb different wavelengths to allow color vision (Figure 1). Cone outer segments are often described as having folds of membrane continuous with the plasma membrane rather than disks separate from the plasma membrane but this is only true in lower vertebrates (Pearring et al., 2013; May-Simera et al., 2017).

Proteins are moved from the site of production, in the inner segment, to the site of light absorption, in the outer segment, along the connecting cilium via a process known as intraflagellar transport (IFT; Ishikawa and Marshall, 2017). The connecting cilium consists of an axoneme of nine microtubule doublets nucleated at the base by a triplet microtubule structure named the basal body. This structure is derived from the mother centriole, at the apical surface of the inner segment. The axoneme extends into the outer segment, converting to singlet microtubules toward the distal end, often reaching near the distal tip of the cone outer segment and at least half-way along the rod outer segment (Roof et al., 1991). The proximal region of the axoneme is stabilized by posttranslational modifications such as glutamylation and acetylation, and is turned over at the distal end as membranes are replaced at the distal end of the outer segment, particularly in cones (Eckmiller, 1996).

Collectively, the connecting cilium and outer segment are termed the photoreceptor cilium, and this is the most highly modified and specialized sensory cilium in the human body (Wheway et al., 2014).

Perhaps unsurprisingly, defects in cilia often lead to retinal phenotypes, either as part of syndromic conditions involving other organs, or in isolation in the so-called retinal ciliopathies (Bujakowska et al., 2017). Non-syndromic retinal ciliopathies include several genetic subtypes of retinitis pigmentosa (RP), Leber Congenital Amaurosis (LCA) and cone-rod dystrophy (CORD).

Retinitis pigmentosa is the most common cause of inherited blindness, affecting up to 1:2000 people worldwide (Golovleva et al., 2010; Sharon and Banin, 2015), and is characterized by night blindness and loss of peripheral vision due to degeneration of rod photoreceptor cells, often progressing to loss of central high acuity vision as cone receptors are also affected (Verbakel et al., 2018). It is normally diagnosed in the third or fourth decade of life, although age of onset and severity vary widely. It can occur in isolation, or as part of syndromes such as Usher syndrome, Bardet Biedl syndrome and Joubert syndrome. The condition is extremely genetically heterogeneous, with 64 genes identified as causes of non-syndromic RP, and more than 50 genes associated with syndromic RP (RetNet^1^) and can be inherited in an autosomal dominant, autosomal recessive, or X-linked manner.

Of the genetic causes of X-linked non-syndromic RP, OFD1, RP2 and RPGR encode cilia proteins. Within autosomal dominant non-syndromic RP (ADRP), RP1 and TOPORS (RP31) encode known cilia proteins. At least 13 genetic causes of autosomal recessive non-syndromic RP (ARRP) encode cilia proteins, including FAM161A (RP28), TTC8 (RP51), C2orf71 (RP54), ARL6 (RP55), MAK (RP62), NEK2 (RP67), BBS2 (RP74), IFT140 (RP80), ARL2BP, RP1L1, C8orf37, CC2D2A and IFT172.

Leber congenital amaurosis is the most common genetic cause of childhood blindness, with an estimated worldwide incidence between 1:33,000 (Alstrom, 1957) and 1:81,000 live births (Stone, 2007). It accounts for 20% of eye disease in children attending schools for the blind (Schappert-kimmijser et al., 1959). Patients with LCA are born with severe visual impairment which is normally diagnosed within a few months of birth by greatly reduced or non-recordable electroretinogram results. Alongside poor vision is nystagmus (involuntary movement of the eyes) and slow to no pupillary response. Although born with already poor eyesight, some patients with LCA undergo further deterioration of vision in adult life, with retinal pigmentary changes often occurring later in life (Heher et al., 1992). It can occur in isolation, or as part of syndromes such as Senior-Loken syndrome. LCA is also genetically heterogeneous, with 13 known genes associated with autosomal recessive LCA, and one gene associated with autosomal dominant LCA (RetNet see footnote 1).

Five genetic subtypes of LCA are known retinal ciliopathies. *LCA5* encodes lebercilin, a ciliary transport protein (den Hollander et al., 2007), *LCA6* encodes RPGRIP1, a ciliary transition zone protein (Dryja et al., 2001), *LCA10* encodes CEP290, a transition zone protein which is also mutated in numerous syndromic ciliopathies (den Hollander et al., 2006) and *LCA15* encodes IQCB1/NPHP5 which interacts with CEP290, localizes to the transition zone and is required for outer segment formation (Estrada-Cuzcano et al., 2010; Ronquillo et al., 2016). All of these proteins localize to the connecting cilium of photoreceptor cells. CLUAP1 (IFT38) is also a cause of LCA (Soens et al., 2016), and plays a central role in photoreceptor ciliogenesis (Lee et al., 2014).

Cone-rod dystrophies (CRD) are rare degenerative conditions with an estimated incidence of 1:40,000 (Hamel et al., 2000). The condition is characterized by loss of cone photoreceptors, leading to loss of central, high acuity vision, disruption of color vision (dyschromatopsia) and photophobia, sometimes followed by degeneration of rod photoreceptors, causing night blindness and tunnel vision. It is normally diagnosed in the first decade of life (Hamel, 2007). It can occur as an isolated condition or as part of the syndromic ciliopathy Alström syndrome (Hearn et al., 2002; Collin et al., 2012). CRDs are also genetically heterogeneous, with 16 autosomal recessive and five autosomal dominant genes having been identified as causing CRD (see footnote 1). Of these, at least seven encode cilia proteins (RAB28 (CORD18), C8orf37 (CORD16), CEP78, POC1B, IFT81, RPGRIP1, and TTLL5).

In total, at least 30 cilia genes have been identified as genetic causes of non-syndromic retinal dystrophies, and this number continues to grow. New ciliary causes of retinal dystrophies continue to be discovered, and new links are made between cilia and retinal conditions not previously considered to be retinal ciliopathies. For example, a recent whole genome siRNA knockdown screen in a ciliated cell line identified PRPF6, PRPF8 and PRPF31, known causes of RP, as cilia proteins (Wheway et al., 2015), offering new perspectives on a poorly understood form of RP.

Clearly, the cilium is of central importance to retinal development and function, with defects in large numbers of cilia proteins leading to various inherited retinal dystrophies. Retinal dystrophies remain extremely difficult to treat, with very few, if any, treatment options for the vast majority of patients, with the exception of RPE65, CEP290, and GUY2D gene therapy in LCA (DiCarlo et al., 2018). In order for this situation to improve, better understanding of the cell biology and molecular genetics of retinal dystrophies, including retinal ciliopathies, is required.

This requires robust, easily genetically manipulated cell models of retinal cells. ARPE19 (ATCC CRL-2302; Dunn et al., 1996), a spontaneously arising male retinal pigment epithelial cell line, and hTERT RPE-1 (ATCC CRL-4000) an hTERT immortalized female retinal pigment epithelial cell line are routinely used in molecular biology studies of retinal ciliopathies, owing to their retinal origin and ability to ciliate upon serum starvation and during late G1 (Spalluto et al., 2013). However, these cells are morphologically and functionally different from photoreceptors, and a dedicated photoreceptor cell line would be of enormous value in this area. Induced pluripotent stem cells (iPSCs) can be reliably differentiated into retinal photoreceptor cells following a 60-day differentiation protocol (Mellough et al., 2012). However, rapid loss of cells committed to a photoreceptor fate (CRX^+^/OPSIN^+^/RHODOPSIN^+^) is seen over days 45–60. The same phenomenon is observed in retinal progenitor cells from mice (Mansergh et al., 2010).

As an alternative to this, optic cups derived from mouse embryonic stem cells (Eiraku et al., 2011), human embryonic stem cells (Nakano et al., 2012) and human iPSCs (Meyer et al., 2011; Reichman et al., 2014) have become popular 3D models for retinal research. These take just 14–18 days to differentiate, and self-assemble in non-adherent culture. The resultant structures are homologous to embryonic retinal cups seen in vertebrate eye development, and include photoreceptors, but are not ideal models of mature retina. These also present problems associated with epigenetic effects, and the spheroid nature of optic cups which prevents access to the center of these organoids for testing or analysis. Attempts to grow and differentiate ESCs and retinal progenitor cells into retinal sheets in custom matrices have been successful, but showed poor lamination (Worthington et al., 2016; Singh et al., 2018).

661W is an immortalized cone photoreceptor cell line derived from the retinal tumor of a mouse expressing SV40 T antigen (Tan et al., 2004). These cells have largely been used as a cell model for studying photo-oxidative stress and apoptosis, but not for studying inherited retinal dystrophies.

Here, we characterize the 661W cell line as a model for studying retinal ciliopathies.

## Materials and Methods

### Cell Culture

661W cells (Tan et al., 2004) were the kind gift of Prof Muayyad Al-Ubaidi, University of Houston. Cells were cultured in DMEM high glucose + 10% FCS at 37°C, 5% CO_2_, and split at a ratio of 1:5 once per week. hTERT-RPE1 cells (ATCC CRL-4000) were cultured in DMEM/F12 (50:50 mix) + 10% FCS at 37°C, 5% CO_2_, and split at a ratio of 1:8 once per week.

### Immunocytochemistry

Cells were seeded at 1 × 10^5^ per mL on sterile glass coverslips in complete media, and after 48 h media was changed to serum-free media, and cells grown for a further 72 h. Cells were rinsed in warm Dulbecco’s phosphate buffered saline (DPBS) and fixed in ice-cold methanol at –20°C for 5 min. Cells were then immediately washed with PBS, and incubated with blocking solution (1% w/v non-fat milk powder/PBS) for 15 min at room temperature. Coverslips were inverted onto primary antibodies in blocking solution in a humidity chamber and incubated at 4°C overnight. After three washes with PBS, cells were incubated with secondary antibodies and DAPI for 1 h at room temperature in the dark. After three PBS washes and one dH_2_O wash, cells were mounted onto slides with Mowiol.

### Antibodies

#### Primary Antibodies for IF

Mouse anti Polyglutamylated tubulin (GT335) 1:1000.
Adipogen Life Sciences AG-20B-0020.
Rabbit anti Gamma-tubulin 1:500. Abcam ab11317.
Rabbit anti Arl13b 1:500. Proteintech 17711-1-AP.
Rabbit anti Ift88 1:500. Proteintech 13967-1-AP.
Rabbit anti Rpgrip1l 1:100. Proteintech 55160-1-AP.
Mouse anti Cep164 1:100. Santa Cruz sc-515403.

#### Secondary Antibodies for IF

Goat anti rabbit IgG AlexaFluor 488 1:1000.
Goat anti mouse IgG AlexaFluor 568 1:1000.
Donkey anti mouse IgG AlexaFluor 488 1:500.
Donkey anti rabbit IgG AlexaFluor 568 1:500.

#### Primary Antibodies for WB

Mouse anti beta actin clone AC-15. 1:4000. Sigma-Aldrich A1978.
Rabbit anti Ift88 1:500. Proteintech 13967-1-AP.

#### Secondary Antibodies for WB

Donkey anti mouse 680 1:20,000 (LiCor).
Donkey anti rabbit 800 1:20,000 (LiCor).

### High Resolution Confocal Imaging

Confocal images were obtained at the Centre for Research in Biosciences Imaging Facility at UWE Bristol, using a HC PL APO 63x/1.40 oil objective CS2 lens on a Leica DMi8 inverted epifluorescence microscope, attached to a Leica SP8 AOBS laser scanning confocal microscope with four solid state AOTF supported lasers (405 nm/50 mW, 488 nm/20 mW, 552 nm/20 mW, and 638 nm/30 mW), two standard PMTs and two high sensitivity HyD hybrid SMD GaAsP detectors. Images were captured using LASX software with Hyvolution II, with automated settings for highest resolution imaging, with pinhole set at 0.5 AU. Images were deconvolved using Huygens Classic Maximum Likelihood Estimation (CMLE) algorithm (Scientific Volume Imaging). Images were assembled in Adobe Photoshop, and figures prepared using Adobe Illustrator.

### Manual Cilium Counting and Length Measurements

Cells immunostained with Arl13b, which labels a larger length of the cilium than our alternative marker, GT335, were imaged at x63, and three fields of view taken to count number of whole nuclei (nuclei at edges of fields of view excluded) and number of cilia. Cilium length was measured using the scale bar as reference. Mean cilium length and percentage of ciliated cells was calculated per experiment. This was repeated in five independent biological replicates. Mean percentage of ciliated cells, and mean cilium length were calculated from the means of all experiments.

### High-Throughput Confocal Imaging

Images were obtained at Wolfson Bioimaging Facility University of Bristol, using the Perkin Elmer OperaLX high-throughput imager, using a 60× water objective lens, 405, 488, and 561 nm lasers. PerkinElmer Opera Adjustment Plate containing multicolour beads was used to define the skewcrop and reference parameters. Cells were grown, fixed and immunostained in 96-well optical bottomed Cell Carrier plates (Perkin Elmer), and images were captured using OperaDB software. Individual z slices were exported as.flex files and assembled into maximum intensity projection z-stacks in Fiji ImageJ (Schindelin et al., 2012). These z-stacks were exported as 16-bit TIFFs, which were imported into CellProfiler for analysis using custom analysis protocols (Carpenter et al., 2006; Kamentsky et al., 2011). Alternatively, z-stacks were constructed in OperaDB, and images analyzed using PerkinElmer Acapella scripts, including algorithms “Find nuclei,” “Find cytoplasm,” and “Find spots” (to find cilia).

### SDS-PAGE and Western Blotting

Total protein was extracted from cells using NP40 lysis buffer and scraping. Insoluble material was pelleted by centrifugation at 10,000 × *g* for 5 min and the total protein concentration of the supernatant was assayed using detergent compatible protein assay kit (BioRad). 20 μg of total protein per sample was mixed with 2 × SDS loading buffer, boiled at 95°C for 5 min, and loaded onto pre-cast 4–12% NuPAGE Novex Bis-Tris gels (Life Technologies) alongside Spectra Multicolor Broad Range Protein ladder (Thermo Fisher). Samples were separated by electrophoreses in MES-SDS running buffer (Life Technologies) at 200 V for 45 min. Protein was transferred to PVDF membrane using wet transfer at 40 V for 2 h. Membranes were incubated with 5% (w/v) non-fat milk/PBS to saturate non-specific binding, and incubated with primary antibody overnight at 4°C. After washing in PBST, membranes were incubated with secondary antibody for 1 h at room temperature and exposed using 680 nm and/or 780 nm laser, or membrane was incubated with SuperSignal West Femto reagent (Pierce) and exposed using Chemiluminescence settings on LiCor Odyssey imaging system (LiCor).

### Scanning Electron Microscopy (SEM)

Scanning electron microscopy images were obtained using a FEI Quanta FEG 650 field emission SEM at UWE Bristol Centre for Research in Biosciences Imaging Facility, using 20 μs dwell, 30.00 kV HV, 3.45 μm HFW. 8.33e–7 Torr pressure, 60,000× magnification.

### RNA Sequencing

Total RNA was extracted from tissue using TRI reagent (Sigma-Aldrich). RNA samples were treated with a TURBO DNA-free^TM^ Kit (Ambion Inc.) using conditions recommended by the manufacturers, and then cleaned with an RNA Clean & Concentrator^TM^-5 spin column (Zymo Research Corp.). RNA was tested for quality and yield using a NanoDrop 1000 spectrophotometer and an Agilent 2100 Bioanalyzer.

Six total RNA samples were supplied and prepared into sequencing libraries from ∼500 ng by Bristol Genomics Facility using the Illumina TruSeq Stranded mRNA kit. Briefly, RNA was polyA-selected, chemically fragmented to about 200 nt in size (4-min fragmentation time), and cDNA synthesized using random hexamer primers. Each individual library received a unique Illumina barcode.

Quality of starting total RNA (diluted 1:100 to be within the limits of the assay) and the final libraries were also assessed using the Agilent TapeStation.

RNA-seq was performed on an Illumina NextSeq500 instrument with six libraries multiplexed and run across four lanes per flow-cell using 75 bp single end reads in high output mode. This resulted in more than 400 million reads per flow cell (651Mill, 590Mill PF), with an average of 94 million reads per sample.

Raw reads from four lanes per sample (four FASTQ files) were aligned to the mouse (*Mus musculus*) full genome (GRCm38, UCSC mm10) using STAR, a splice-aware aligner (Dobin et al., 2013), with UCSC mm10.gtf gene model for splice junctions and the resultant BAM files were merged.

Again, using UCSC mm10.gtf file, raw gene counts were estimated on merged BAM files using HTSeq, using the union method and –stranded = reverse options (Anders et al., 2015). Differential gene expression was analyzed using DESeq2 (Love et al., 2014) with statistical significance expressed as a *p*-value adjusted for a false discovery rate of 0.01 using Benjamini-Hochberg correction for multiple-testing.

Gene ontology (GO) Enrichment Analysis was carried out on the genes found to be differentially expressed between unstarved and starved cells using DAVID (Huang da et al., 2009).

For comparison to hTERT-RPE1 cell expression, we downloaded RNA sequence data from untreated and serum starved hTERT-RPE1 cells from the Sequence Read Archive (SRA; SRR2895378 and SRR2895380). These samples were selected for comparison to our 661 W samples, because the same protocols were followed for serum starvation, RNA extraction and purification, library preparation and sequencing. However, the sequencing read length for hTERT-RPE1 samples was shorter than for 661 Ws (50 bp single-end reads) and at lower depth (21 million reads and 26 million reads per sample). This data has been previously published in Wheway et al. (2015). Raw hTERT-RPE1 reads were aligned to the human (*Homo sapiens*) full genome (GRCh38.92, UCSC hg38) using STAR (Dobin et al., 2013), with UCSC hg38.gtf gene model for splice junctions.

Cufflinks tool (Trapnell et al., 2012) was used to calculate transcripts per kilobase of exon per million reads mapped (TPKM) for all assembled transcripts for all 661W samples and hTERT-RPE1 samples, and comparisons made between genes of interest between hTERT-RPE1 cell expression and 661W cell expression.

## Results

**FIGURE 2:**
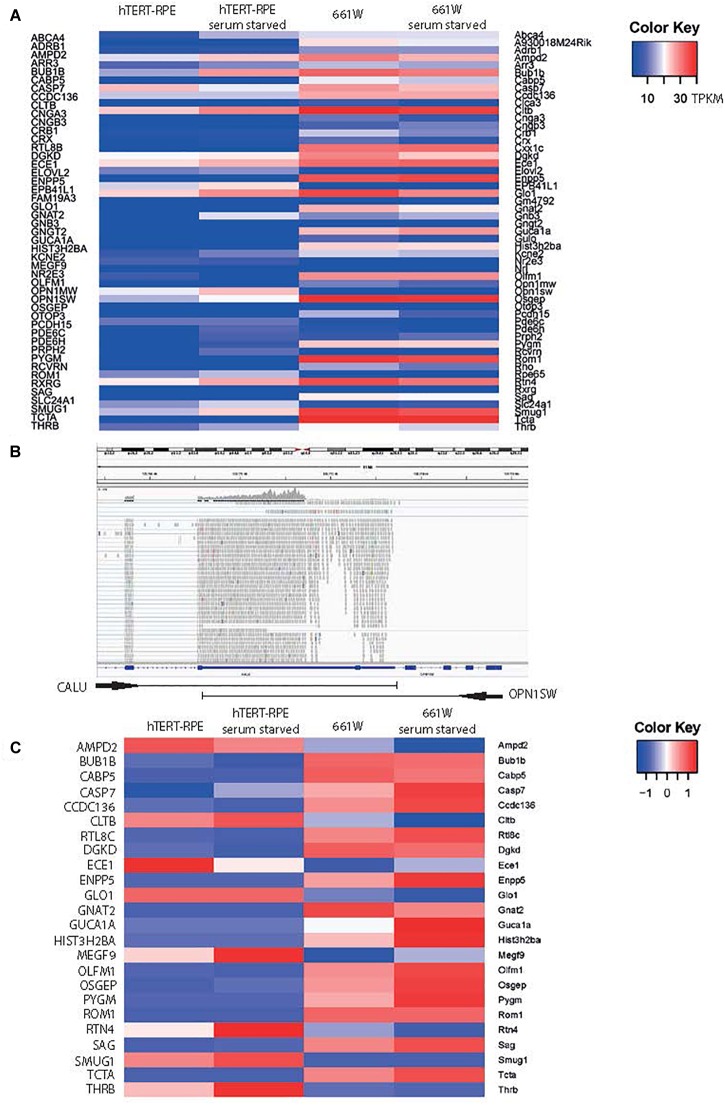
661W cells express markers of photoreceptor fate. **(A)** Heat map of expression of 48 genes characteristic of cone photoreceptors (Corbo et al., 2007) in hTERT-RPE1 cells and 661W cells in conditions of serum and serum starvation. Scale bar shows intensity of color relative to TPKM. **(B)** IGV screenshot of OPN1SW genomic location in human genome hg38, with one shared exon with CALU, which accounts for the mapping of reads to OPN1SW in the hTERT-RPE1 cell line. **(C)** Heat map showing expression level of all cone cell genes expressed in 661W cells, relative to the expression these genes in hTERT-RPE1 cells. Scale bar shows intensity of color relative to scaled expression value from –1 to 1 across each row.

To confirm the cone photoreceptor origin of this cell line, and to fully characterize the expression profile of these cells, we performed whole transcriptome RNA sequencing on 661W cells, with and without exposure to serum starvation (GEO Accession: GSE119190; SRA Accession: SRP159075) and compared these to expression in hTERT-RPE cells subjected to the same growth conditions (SRA Accession: SRR2895378 and SRR2895380; Supplementary Table 1). Cells were starved of serum for 72 h to induce the cells to exit the cell cycle and form post-mitotic cilia (Santos and Reiter, 2008). We filtered the data to specifically study 47 genes which show characteristic expression in cone photoreceptor cells (Sharon et al., 2002; Corbo et al., 2007; Figure 2A and Supplementary Table 2). The data shows that 661W cells express a range of cone markers. Surprisingly, our data seemed to suggest that OPN1SW was expressed in the hTERT-RPE1 cells with a TPKM of around 4, but analysis in Integrative Genomics Viewer (IGV; Robinson et al., 2011), showed that this was based upon alignment of many reads to one exon of OPN1SW which is shared with the neighboring gene CALU, which is highly expressed in these cells (Figure 2B). Based on our analysis of other alignments in IGV we decided to exclude any gene with an abundance estimate of less than 0.1 TPKM, and consider these as being not expressed. Figure 2C shows all markers of cone cell origin which are expressed in 661W cells, but are generally not expressed in hTERT-RPE1 cells, including cone alpha transducin (Gnat2) and Rom1. The data suggests that expression of most of these genes increase upon serum starvation, suggesting that the cells are induced toward a more cone-like fate upon serum starvation (Figure 2C). Short wave and medium wave opsin (Opn1sw and Opn1mw) and cone arrestin (Arr3) were not expressed in either cell type. This is in contrast to previously published papers showing expression of these cone markers in the 661W cell line. Earlier publications studied early passages of this cell type, whereas we studied cells between passage 24 and 27. This suggests that expression of these cone markers is not sustained over many passages.

**FIGURE 3:**
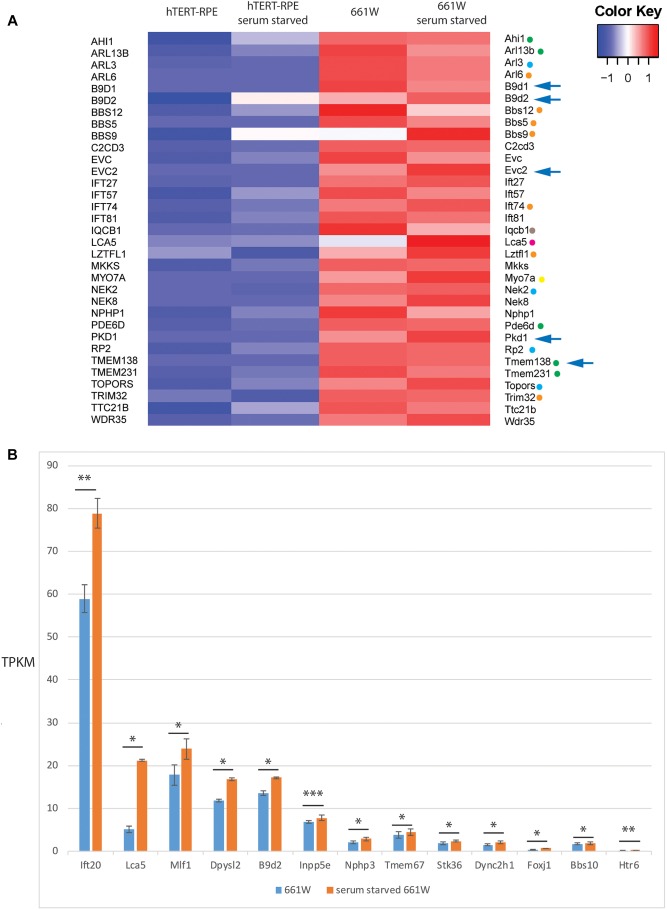
661W cells express markers of ciliated cell fate. **(A)** Heat map showing expression level of 33 Syscilia Gold Standard (SCGS) genes expressed in 661W cells which are either not expressed in hTERT-RPE1 cells, or are expressed at a low level in hTERT-RPE1 cells. Green dots indicate Joubert syndrome disease genes, blue dots indicate retinitis pigmentosa disease genes, orange dots indicate Bardet-Biedl syndrome disease genes, borwon dot indicates Senior-Loken syndrome disease gene, pink dot indicates Leber congenital amaurosis disease gene, yellow dot indicates Usher syndrome disease gene. Arrows indicate syndromic disease genes which are expressed in 661W cells but not expressed at all in hTERT-RPE1 cells. **(B)** Graph showing mean TPKM of selected SCGS genes in unstarved and serum starved cells, to show upregulation of these genes upon serum starvation to induce cilium formation. ^∗∗∗^*p* < 0.005; ^∗∗^*p* < 0.01; ^∗^*p* < 0.05.

To investigate expression of cilia genes in this cell line, we extracted TPKM transcript abundance estimates data from our RNAseq data for Syscilia Gold Standard (SCGS) genes (van Dam et al., 2013) in starved and unstarved hTERT-RPE1 and 661W cells. Again, excluding any genes with an abundance estimate of less than 0.1 TPKM, the 661W cells showed robust expression of 213 SCGS genes in unstarved and starved cells, including markers such as Cep164, Rpgrip1l and Ift88 (Supplementary Table 3). The cells also expressed numerous alpha- beta- and gamma-tubulin genes, as well as enzymes involved in post-translational modification of ciliary axonemal microtubules, such as Ttll9, which is involved in polyglutamylation of tubulin. Furthermore, 661W cells expressed 33 genes which are either not expressed in hTERT-RPE1 cells, or expressed at a low level in hTERT-RPE1 cells (Figure 3A). This includes many disease genes associated with a retinal ciliopathy phenotype, including Joubert disease genes Ahi1, Arl13b, Pde6b, Tmem138, and Tmem231; Bardet-Biedl disease genes Arl6, Bbs12, Bbs5, Bbs9, Ift74, Lztfl1, and Trim32; Senior-Loken syndrome gene Iqcb1; retinitis pigmentosa disease genes Arl3, Nek2, Rp2, and Topors; Leber congenital amaurosis disease gene Lca5 and; Usher syndrome gene Iqcb1 (Figure 3A). In addition to this, 661W cells express several syndromic ciliopathy disease genes which are not expressed at all in hTERT-RPE1 cells, including B9d1, B9d2, Evc2, Pkd1, and Tmem138 (Figure 3B). For the study of these disease genes, 661W cells are a valuable resource, as studies in the widely used retinal cell line hTERT-RPE1 will not be effective.

Of the 213 SCGS genes expressed in 661W cells, there is a statistically significant (*p* < 0.05) up or down regulation of 22/213 (10.3%) of these genes upon serum starvation. suggesting that the cells are induced toward a more differentiated state upon serum starvation (Figure 3B).

We then used HTSeq and DESeq2 to identify all genes differentially expressed in different conditions (starved vs. unstarved), controlling for batch-effects, and performed enrichment analysis on all hits with an adjusted *p*-value of >0.01 using DAVID (Huang da et al., 2009; Supplementary Table 4). This identified a number of enriched annotation clusters, including those with the GO terms “neuron differentiation” (enrichment score 2.22), “eye development” (enrichment score 0.16) and “sensory perception” (enrichment score 0.52), “GPCR, rhodopsin-like superfamily” (enrichment score 0.25) and “centrosome” (enrichment score 0.010; Supplementary Table 4).

**FIGURE 4:**
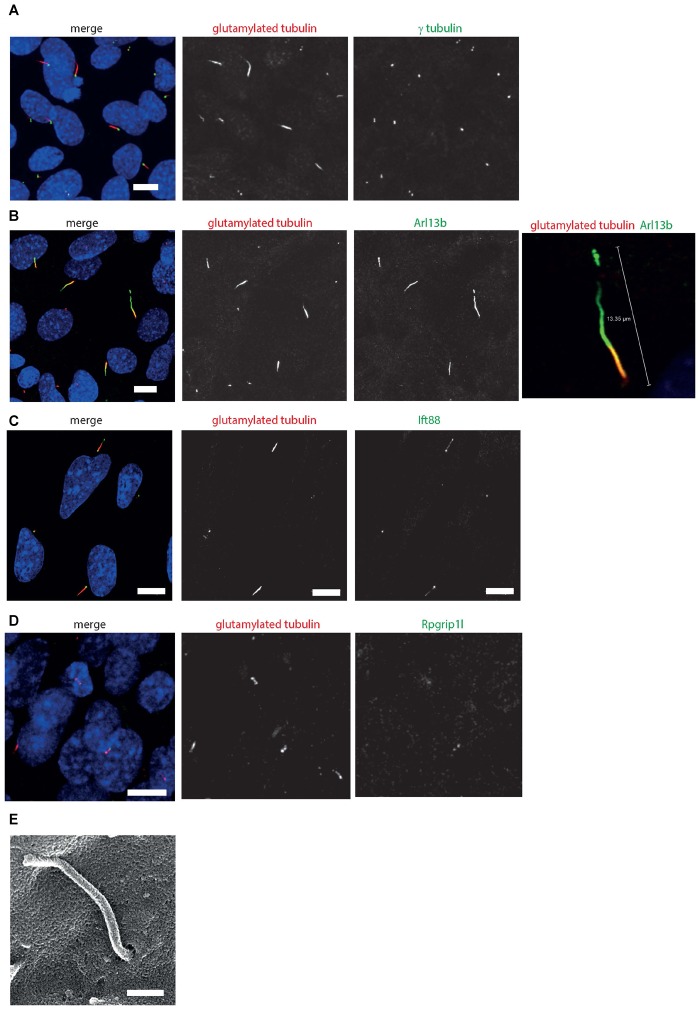
661W cells grow long primary cilia. **(A)** 661W stained with proximal axonemal marker polyglutamylated tubulin (red), basal body marker γ tubulin (green) show that many cells grow primary cilia. Cells counterstained with DAPI (blue). Scale bar 10 μm. **(B)** Staining with cilium membrane marker Arl13b (green) shows that these cells grow cilia up to approx. 15 μm long, with only the proximal portion of the axoneme polyglutamylated. Scale bar 10 μm **(C)** Staining with Ift88 antibody (green) shows that this IFT protein localizes along the cilium, with more concentrated localisation at the base and tip of the cilium. Scale bar 10 μm. **(D)** Staining with Rpgrip1l antibody (green) shows that this transition protein colocalises with polyglutamylated tubulin, with more concentrated localisation at the base of this region of polyglutamylated tubulin. Scale bar 10 μm. **(E)** Scanning electron microscope image of 661 W cell, showing cilium in ciliary pit. Scale bar = 500 nm.

Immunostaining and confocal microscopy of these cells shows that on average, 30.2% of serum starved cells grow a primary cilium (st. error 2.49, *n* = 5 independent replicates), with a mean cilium length of 3.01 μm (st. error 0.45, *n* = 5 independent replicates). The cells localize many cilium proteins to the axoneme (polyglutamylated tubulin, Ift88), ciliary transition zone (Rpgrip1l), cilium membrane (Arl13b) and basal body (gamma tubulin; Figure 4A–D). The cells can grow primary cilia up to almost 15 μm in length (Figure 4B). The ability to form long cilia is consistent with the long axonemes of cones seen *in vivo*. Whilst the axoneme of rod photoreceptors only extends along a portion of the outer segment, it extends along the entirety of the cone outer segment in *Xenopus laevis* toads *in vivo* (Eckmiller, 1996). The axoneme of most primary cilia tend to be extensively post-translationally modified, but in 661W cells only a small portion of the proximal axoneme is polyglutamylated (Figure 4A–D), consistent with a similar observation in cones in mice, which show extensive glutamylation and glycation in the connecting cilium region of the photoreceptor, but none along the axoneme of the outer segment of the cones (Bosch Grau et al., 2017). This level of post-translational modification is tightly regulated in mouse cones, and disruption of glycylation leading to hyperglutamylation leads to retinal degeneration (Bosch Grau et al., 2017). SEM shows these cilia grow in characteristic ciliary pits in the cell membrane (Figure 4E).

**FIGURE 5:**
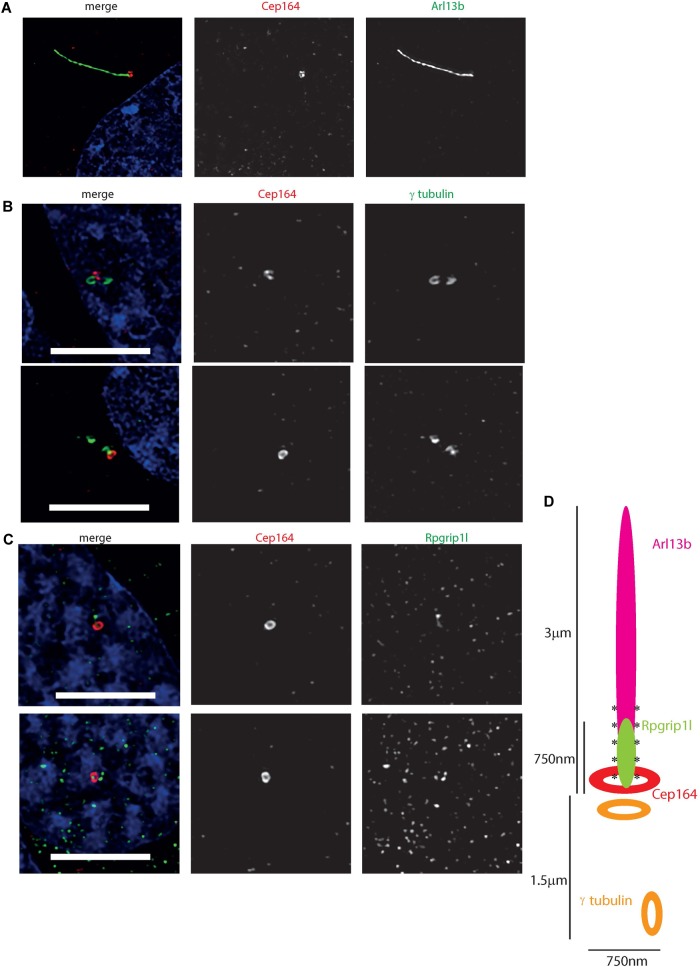
Ultrastructure of the 661 W cilium. **(A)** Hyvolution imaging of distal centriolar appendage marker Cep164 (red), which localizes specifically to the mature mother centriole of the basal body, and cilium membrane protein Arl13b (green). **(B)** Hyvolution imaging of distal centriolar appendage marker Cep164 (red), which localizes specifically to the mature mother centriole of the basal body, and γ tubulin (green) which labels both mother and daughter centrioles. Scale bar = 5 μm. **(C)** Hyvolution imaging of distal centriolar appendage marker Cep164 (red), and transition zone protein Rpgrip1l (green). Scale bar = 5 μm. **(D)** Schematic representation of protein localisation in 661W cilia, with scale bars, labeled. Asterisks symbolize region of polyglutamylation of the axoneme.

To further resolve the ultrastructure of this cilium, we used confocal microscopy with deconvolution to analyze the relative localisation of a centrosomal and basal body marker (gamma tubulin), distal centriolar appendage marker (Cep164), axonemal marker (Arl13b) and transition zone marker (Rpgrip1l) along the cilium (Figure 5A–C). This revealed an Arl13b cilium membrane extending from a ring of Cep164, lying distal to a ring of gamma tubulin of the mother centriole-derived basal body. A second gamma tubulin ring, of the daughter centriole, lies at approximately right-angles to this basal body. Rpgrip1l, a transition zone marker, extends from within the ring of Cep164 beyond this ring, presumably along the axoneme of the cilium. This differs from descriptions of Rpgrip1l localisation within the transition zone in primary cilia, where it is normally seen to reside in a tight ring narrower in diameter to Cep164, but more distal to Cep164 (Yang et al., 2015). However, it is known that transition zone assembly is highly cell-type specific, and Rpgrip1l plays a key role in this process (Wiegering et al., 2018). Extension of Rpgrip1l further beyond the distal appendages in this cell line is reminiscent of RPGR1P1L localisation in photoreceptors *in vivo*, where it is localized along the connecting cilium (Dharmat et al., 2018).

Non-coding RNA data from RNA sequencing of this cell line identifies a number of novel RNAs not previously linked to photoreceptor fate (Supplementary Table 5). Most noteworthy is mmu-mir-6236, which was expressed at very high levels in starved and unstarved cells. This miRNA has not been previously linked to photoreceptor fate or function. These cells were also found to express miR-17-hg, which has been previously linked to neuronal differentiation (Bian et al., 2013; Mao et al., 2016), and may contribute to neuronal photoreceptor differentiation in these cells. Interestingly, expression of this microRNA has been shown to promote cyst growth in polycystic kidney disease, a common ciliopathy (Patel et al., 2013). It could be hypothesized that this miRNA plays a role in ciliogenesis in both the kidney and the photoreceptor.

**FIGURE 6:**
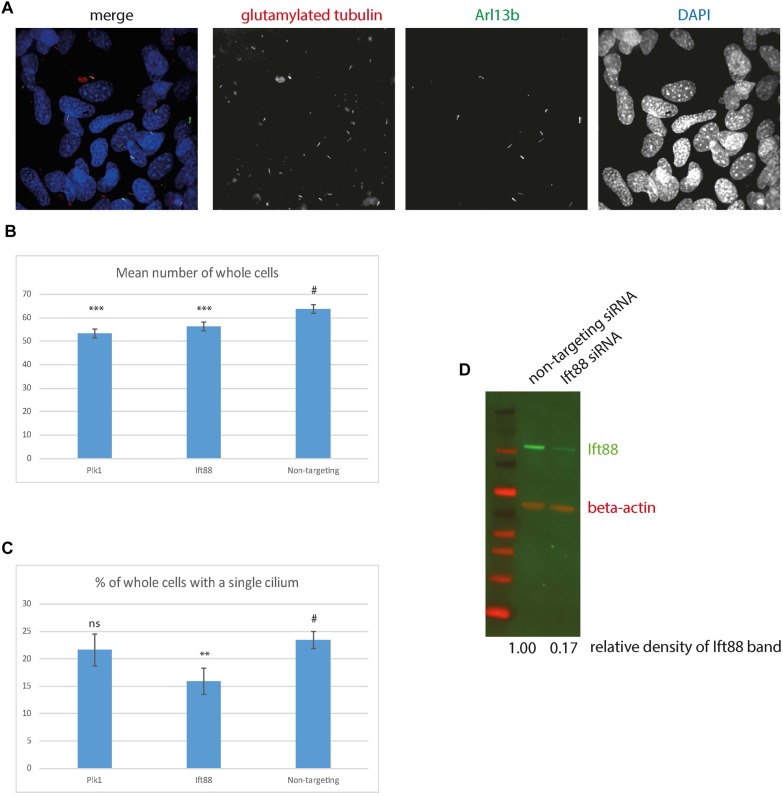
High content imaging of 661 W cells. **(A)** Example images from the Perkin Elmer Opera, showing 661W cells stained with glutamylated tubulin (red), Arl13b (green) and DAPI (blue). **(B)** Mean number of whole cells per field of view is statistically significantly reduced after 72 h Plk1 and Ift88 siRNA knockdown compared to cells treated with non-targeting control, ^∗∗∗^*p* < 0.0001. **(C)** % of cells with a single cilium per field of view is statistically significantly reduced after 72 h Ift88 siRNA knockdown compared to cells treated with non-targeting control, ^∗∗^*p* < 0.001, ^#^ is control experiment to which the statistical significance of the knockdowns are compared. **(D)** western blotting confirms reduction in Ift88 protein level (green) after 72 h Ift88 siRNA knockdown, compared to non-targeting control. Red shows beta actin loading control.

Finally, in order to evaluate the utility of 661 W cells for high-throughput screening, we performed siRNA knockdown of cilia protein Ift88 in 661 W cells in 96 well optical-bottom culture plates, and assayed cell number and cilia number by high-content imaging, using Arl13b and polygutamylated tubulin as cilia markers (Figure 6A). Nuclei were identified using DAPI, and cilia were detected using a modified “find spots” Perkin Elmer image analysis algorithm. This showed that knockdown of positive control Plk1 induced a statistically significant reduction in cell number (Figure 6B), and Ift88 knockdown induced a statistically significant reduction in the number of cells with a single cilium (Figure 6B,C). Western blotting confirmed knockdown of Ift88 (Figure 6D). The automated image analysis showed good agreement with our manual measurements of cilia number; around 22% of cells using manual counting.

## Discussion

Our comprehensive deep sequencing of total mRNA and selected non-coding RNAs in the 661 W cell line confirms its cone photoreceptor origin, and ability to grow a photoreceptor-like cilium in culture after serum starvation (Figure 2–4 and Supplementary Table 1). A recent paper described the 661 W cell line as a retinal ganglion-like cell line, owing to its expression of markers specific to retinal ganglion cells, such as Rbpms, Pouf2, Pouf3, Thy1, and γ-synuclein (Sayyad et al., 2017). Our whole transcriptome RNA sequence data confirms that this cell line expresses Rbpms, Thy1, and Sncg at low levels, but does not express Pouf2 or Pouf3 (Supplementary Table 1). This suggests that this cell line does indeed have some features of retinal ganglion cells, but also expresses markers of cone photoreceptor fate. Our data supports the conclusion of Sayyad et al. (2017), that this cell line shows properties of both retinal ganglion and photoreceptor cells, and is a useful *in vitro* photoreceptor model.

Immunofluorescence confocal microscopy and deconvolution of images of these cells reveals a cilium similar in structure to cone photoreceptor cilia *in vivo*. After serum starvation in culture, around a third of cells extend a cilium of around three microns in length which is only post-translationally modified at the proximal end, and localizes Rpgrip1l along this proximal region (Figure 4, 5). The cell line expresses many *bona fide* cilia genes, including many mutated in retinal ciliopathies, and expression of many of these increases upon serum starvation of the cells (Figure 3 and Supplementary Table 3). Knockdown of key cilia gene Ift88 results in robust reduction in percentage of whole cells with a single cilium, which can be readily assayed by high-content imaging (Figure 6).

Our demonstration of high-content imaging of 661 W cells illustrates the potential utility of this cell line for high-throughput screening. A recent high-throughput small molecule screen in hTERT-RPE1 cells successfully identified eupatilin as a small molecule which rescued transition zone defects in CEP290 knockout RPE1 cells (Kim et al., 2018). A similar approach using 661 W cells could be an even more clinically relevant method for identifying small molecules which could be used for treating retinal ciliopathies. Similarly, the cell line could be useful for reverse genetics functional genomics screening to identify novel retinal cilia genes and retinal ciliopathy candidate genes, similar to previous screens (Wheway et al., 2015). These methods are of enormous importance in a field where effective treatments are so limited.

The 661 W cell line can be particularly useful for studying ciliopathy disease genes which are not expressed in hTERT-RPE1 cells, or are only expressed at a low level in this cell line (Figure 3A).

In summary, we provide evidence that 661 W cells are a useful *in vitro* model for studying retinal ciliopathies.

## Author Contributions

GW conceived the study, performed the experiments, carried out the data analysis, imaging, image analysis, bioinformatics analysis, and wrote the paper. LN performed the experiments, carried out the data analysis, imaging, and image analysis. DT contributed to bioinformatics analysis. SC provided support in high-throughput imaging and image analysis.

## Conflict of Interest Statement

The authors declare that the research was conducted in the absence of any commercial or financial relationships that could be construed as a potential conflict of interest.

## Footnotes

1 https://sph.uth.edu/retnet/sum-dis.htm

## Supplementary Material

TABLE S1 Transcript abundance estimates in transcripts per kilobase of exon per million reads mapped (TPKM) for all genes in all repeats of starved and unstarved 661W cells (tab 1) and hTERT-RPE1 cells (tab 2).

TABLE S2 Transcript abundance estimates in transcripts per kilobase of exon per million reads mapped (TPKM) for 47 cone photoreceptor genes in all repeats of starved and unstarved 661W cells and hTERT-RPE1 cells.

TABLE S3 Transcript abundance estimates in transcripts per kilobase of exon per million reads mapped (TPKM) for 277 Syscilia Gold Standard (SCGS) genes in all repeats of starved and unstarved 661W cells and hTERT-RPE1 cells.

TABLE S4 Results of differential gene expression analysis of starved and unstarved 661W cells (tab 1) and DAVID functional annotation cluster analysis of differentially expressed genes (tab 2).

TABLE S5 Transcript abundance estimates in transcripts per kilobase of exon per million reads mapped (TPKM) for non-coding RNAs in all repeats of starved and unstarved 661W cells.
